# THE GUT MICROBIOME AND AGING

**DOI:** 10.1093/geroni/igz038.3070

**Published:** 2019-11-08

**Authors:** Christy S Carter, Michal Masternak, Thomas W Buford

**Affiliations:** 1 University of Alabama at Birmingham, Birmingham, Alabama, United States; 2 University of Central Florida, Orlando, Florida, United States

## Abstract

The human intestinal tract (i.e., “gut”) is inhabited by over 100 trillion microorganisms; including over 1000 species of known bacteria. These organisms have co-evolved with humans over millennia to live together for mutual benefit. Though long overlooked in considerations of human health and disease treatment, gut microorganisms are highly involved in numerous metabolic reactions which influence normal host physiology. A variety of biologic, medical, and lifestyle factors appear to contribute to gut dysbiosis in late-life, and interventions specifically designed to target these factors may be useful in restoring microbial balance. Evidence from both clinical and preclinical studies suggests that gut dysbiosis is related to age-related inflammation as well as age-related conditions including frailty, Alzheimer’s disease, and perhaps even longevity. Crosstalk between the gut and multiple organ systems (brain, heart, muscle etc.) may lead to the development of age-related diseases and loss of physiological function, although the signals are not well understood. In this symposium we address the broad topic of the Gut Microbiome and Aging by presenting evidence from multiple model systems (mice, rats and monkeys) and provide a forum to discuss critical areas of research for moving forward.

